# Silencing the Autophagy-Related Genes *ATG3* and *ATG9* Promotes SRBSDV Propagation and Transmission in *Sogatella furcifera*

**DOI:** 10.3390/insects13040394

**Published:** 2022-04-18

**Authors:** Dandan Liu, Zhengxi Li, Maolin Hou

**Affiliations:** 1 State Key Laboratory for Biology of Plant Diseases and Insect Pests, Institute of Plant Protection, Chinese Academy of Agricultural Sciences, Beijing 100193, China; liudandan2016@yeah.net; 2 College of Plant Protection, China Agricultural University, Beijing 100193, China; zxli@cau.edu.cn

**Keywords:** *Sogatella furcifera*, southern rice black-streaked dwarf virus, autophagy-related genes, virus transmission

## Abstract

**Simple Summary:**

Plant viruses are mostly transmitted by insects and cause severe damage to crops. The southern rice black-streaked dwarf virus is a rice virus exclusively transmitted by a planthopper, *Sogatella furcifera*. Autophagy is usually initiated as an adaptive response for cell survival in unfavorable conditions, such as pathogen invasion. The invasion of the virus in the vector may elicit autophagy and may subsequently function to suppress or promote virus propagation and transmission in the insect vector. Here, we annotated two key autophagy-related genes, *SfATG3* and *SfATG9*, in the planthopper vector and found that the genes are conserved among various insects. Interestingly, exposure of the vector to the virus activated the expression of *SfATG3* and *SfATG9*, and silencing *SfATG3* or *SfATG9* promoted propagation and transmission rates of the virus. These results indicate that the expression of autophagy-related genes is initiated in the vector following exposure to the virus and that autophagy-related genes functions to suppress SRBSDV propagation and transmission.

**Abstract:**

Autophagy plays diverse roles in the interaction among pathogen, vector, and host. In the plant virus and insect vector system, autophagy can be an antiviral/pro-viral factor to suppress/promote virus propagation and transmission. Here, we report the antiviral role of autophagy-related genes *ATG3* and *ATG9* in the white-backed planthopper (*Sogatella furcifera*) during the process of transmitting the southern rice black-streaked dwarf virus (SRBSDV). In this study, we annotated two autophagy-related genes, *SfATG3* and *SfATG9*, from the female *S. furcifera* transcriptome. The cDNA of *SfATG3* and *SfATG9* comprised an open reading frame (ORF) of 999 bp and 2295 bp that encodes a protein of 332 and 764 amino acid residues, respectively. *SfATG3* has two conserved domains and *SfATG9* has one conserved domain. In *S. furcifera* females exposed to SRBSDV, expression of autophagy-related genes was significantly activated and shared similar temporal patterns to those of SRBSDV S9-1 and S10, all peaking at 4 d post viral exposure. Silencing the expression of *SfATG3* and *SfATG9* promoted SRBSDV propagation and transmission. This study provides evidence for the first time that *S. furcifera* autophagy-related genes *ATG3* and *ATG9* play an antiviral role to suppress SRBSDV propagation and transmission.

## 1. Introduction

The survival, transmission, and spread of vector-borne viruses depend on the interplay among insect vector, host, and virus [1,2]. Insect vector transmits the virus in a non-persistent or persistent manner. The persistently transmitted viruses initiate propagation after they are ingested with the phloem sap of infected plants [1,3]. Later, virions break the limits of intestinal epithelial cells of insects and disseminate from hemocoel into salivary glands [4]. Finally, virions are horizontally transmitted to healthy plants with the saliva [1,4,5]. Such a process of virus circulation in insects induces a series of cellular responses, such as autophagy [6,7,8,9].

Autophagy is an important innate cellular immune response in eukaryotes. It is a highly conserved catabolic process that mediates the clearance of long-lived proteins and damaged organelles via a lysosomal degradative pathway [10,11,12,13,14,15]. Autophagy induced by virus infection may function to inhibit virus propagation in vectors, as that reported in the tomato yellow leaf curl virus (TYLCV)-the whitefly (*Bemisia tabaci*) vector system, where autophagy activated by TYLCV infection led to the degradation of viral particles and suppressed virus transmission [16]. In rice black-streaked dwarf virus (RBSDV)- the small brown planthopper (*Laodelphax striatellus*) system, autophagy induced by RBSDV infection in *L.* *striatella* resulted in suppression of RBSDV invasion and propagation [17]. Autophagy can, on the contrary, promote virus transmission in insect vectors. Autophagy activated in the leafhopper vector (*Recilia dorsalis*) by infection of two plant viruses, rice gall dwarf virus (RGDV) and rice dwarf virus (RDV), facilitated viral propagation and transmission [18]. Besides, autophagic activity in *Laodelphax striatellus* did not directly affect the propagation or transmission of the rice stripe virus (RSV), but increased the phosphorylation of c-Jun *N*-terminal kinase [19], which facilitated the RSV propagation and transmission [20].

Many genes participate in autophagy for promoting or inhibiting pathogens, for example, autophagy-related genes (ATGs). Under normal circumstances, autophagy keeps at a low level, but it can be activated by stress signals such as pathogens, which are known to induce the expression of some ATGs [21]. Numbers of ATGs are identified from potato psyllid, *Bactericera cockerelli*, whose expression is activated by pathogenic infection [22]. Thus, the identification of genes that participates in autophagy will deepen our understanding of the role of autophagy in different physiological processes.

The southern rice black-streaked dwarf virus (SRBSDV) causes severe yield losses in Asia [23]. The viral genome contains ten linear double-stranded RNA (dsRNA) segments that range in size from approximately 4.5 to 1.8 kb and are named S1–S10 according to their molecular weights [23]. The virus is exclusively transmitted by the white-backed planthopper (WBPH) [24,25]. Studies have revealed a complex interaction pattern among rice plant, SRBSDV, and WBPH. Compared with WBPH fed on non-infected rice, WBPH fed on infected rice showed significantly prolonged longevity but notably decreased fecundity [26,27]. In addition, viruliferous WBPH fed on healthy plants spent more time in salivation and phloem sap ingestion than non-viruliferous insects [28], which may enhance virus transmission. Further transcriptome sequencing studies demonstrated that SRBSDV infection activated the expression of WBPH ATGs [29,30]. However, it is not clear how the role of ATGs when WBPH interacts with SRBSDV transmission.

Given the varying roles of vector autophagy in virus transmission, the present study was conducted to determine the role of ATGs in the transmission of SRBSDV.ATG3 catalyzes the conjugation of ATG8 and phosphatidylethanolamine, APG9 (autophagy-related protein 9 in mammalians, which is the same as ATG9 in other eukaryotes) is the only integral membrane ATG protein that is essential for autophagosome formation. Therefore, as a start point, we annotated *ATG3* and *ATG9* from the WBPH transcriptome data and analyzed the sequences of *SfATG3* and *SfATG9.* The expression patterns of autophagy-related genes and SRBSDV genomes were detected in WBPH females exposed to SRBSDV in comparison to those exposed to healthy rice plants by qPCR. Finally, the role of autophagy-related genes in SRBSDV propagation, acquisition, and inoculation was determined by silencing *SfATG3* and *SfATG9*.

## 2. Materials and Methods

### 2.1. Insects and Plants

Potted seedlings of rice (var. Taichung Native 1, TN1) were cultured in 80-mesh cages (50 by 50 by 50 cm) in a greenhouse (30 ± 5 °C, 15L:9D). A WBPH population was maintained using caged rice seedlings (var. TN1). SRBSDV originated from rice seedlings collected from Xing’an (25°36′18″ N, 110°42′16″ E, Guangxi Province, China), and a virus stock culture was maintained in caged rice plants in the greenhouse.

To obtain SRBSDV-infected rice plants, SRBSDV-free 30 d old rice seedlings were caged with viruliferous WBPHs at a density of five or seven insects per rice for 5 d. Fifteen days thereafter, the SRBSDV infection status of these rice seedlings was detected individually by one-step RT-PCR based on SRBSDV S10 [31]. SRBSDV-positive plants were maintained in insect-proof cages in the greenhouse for subsequent use in the experiments.

### 2.2. Bioinformatics Analysis

Due to their indispensable function of *ATG3* and *ATG9* coding genes in autophagy, we identified *SfATG3* and *SfATG9* coding genes from female WBPH transcriptome data (Accession number: PRJNA781429). The deduced amino acid sequences of *SfATG3* and *SfATG9* genes were obtained using the Translate tool provided by the Swiss Institute Bioinformatics (https://web.expasy.org/translate/, accessed on 23 March 2022). Domain analysis of the retrieved protein sequence was executed by NCBI Conserved-Domain Tool (https://www.ncbi.nlm.nih.gov/cdd, accessed on 23 March 2022) and Prosite (https://prosite.expasy.org/, accessed on 23 March 2022) to predict the conserved domains in the open reading frame (ORF) of *SfATG3* and *SfATG9.* The phylogenetic relationship of ATG3 between WBPH and 14 other species and of ATG9 between WBPH and 9 other species were established by constructing a phylogenetic tree using MEGA7.0, where phylogenetic analysis was conducted by the neighbor-joining method with P-distance under the default parameters. Bootstrap values were obtained by the bootstrap method using 5000 repetitions. The species name, gene name, and GenBank accession involved in the phylogenetic analysis were listed in Tables S1 and S2.

### 2.3. Temporal Expression of Virus Gene and Autophagy-Related Genes in SRBSDV-Exposed WBPH Females

Newly emerged 500 WBPH females were fed on SRBSDV-infected rice seedlings for 2 d for virus acquisition, which resulted in more than 80% of WBPH females being SRBSDV-positive, and then the females were transferred to healthy plants. At 0, 2, 4, 8, or 12 days post-exposure (dpe), 30 females were randomly sampled for 3 duplicates to determine the temporal patterns of viral infection influence on the expression of several key autophagy-related genes *SfATG3* and *SfATG9* and SRBSDV genomes, including the first part of the 9th RNA segment of the genome (52–1095 nt) that encodes a key viroplasm protein and the 10th RNA segment of genome that encodes the structural protein (S9-1 and S10, respectively).

To determine the expression of *SfATG3* and *SfATG9*, total RNA was extracted from ten WBPH females using the Total RNA Extraction Kit (Solarbio Science & Technology, Beijing, China) and reverse-transcribed into cDNA using PrimeScript RT Master Mix (both Oligo dT primer and random 6 mers primer were used in the same reaction, Takara Biomedical Technology, Beijing, China). Besides, the SRBSDV genomic RNA abundance was also measured with S9-1 and S10 to determine the propagation of the virus in WBPH. The housekeeping gene elongation factor 1-α (*EF1α*) of WBPH was measured in parallel as an internal control [32]. Real-time quantitative PCR was performed with the primer sequences shown in Table 1 on the ABI 7500 Real-Time PCR system (NanoDrop Technologies, Wilmington, USA).

### 2.4. SRBSDV Transmission by WBPH Treated with dsRNAs

#### 2.4.1. Synthesis of dsRNA

Specific primers (Table 1) were designed to synthesize ds*ATG3*, ds*ATG9,* and ds*GFP* using the T7 transcription Kit (Tiandz. Inc., Beijing, China) according to the manufacturer’s instructions. The size of dsRNA products was confirmed by electrophoresis in 1% (*w*/*v*) agarose gels (Figure S1), and the final concentration of dsRNA was adjusted to 400 ng/μL.

#### 2.4.2. SRBSDV Acquisition and Expression of Virus Gene and Autophagy-Related Genes

Newly emerged SRBSDV nonviruliferous WBPH females were fed with dsRNA (ds*ATG3* and ds*ATG9*) mixed with 15% sucrose (*v/v* = 50/50) in a 50-mm diameter cylindrical container for 24 h. WBPH females fed with ds*GFP* were used as the control for those insects fed with dsRNAs. Then the insects were confined with SRBSDV-infected rice seedlings for 2 d and thereafter transferred to healthy rice plants. At 5 dpe, the insects were individually detected for SRBSDV infection status. The test of SRBSDV acquisition was performed on a batch of more than 15 females and repeated in three batches for treatment or control. The SRBSDV acquisition rate was calculated as the number of viruliferous insects in a batch/number of tested insects in the batch.

Expression of SRBSDV S9-1 and S10 and autophagy-related genes in each treatment and control was measured to 10 randomly sampled WBPH females at 5 dpe using the qPCR procedure as described above. The measurement was repeated three times for each treatment and control.

#### 2.4.3. SRBSDV Inoculation

Newly emerged nonviruliferous WBPH females were fed with dsRNAs for 24 h as above and then confined to SRBSDV-infected rice plants for 2 d for virus acquisition and thereafter transferred to healthy rice plants and maintained for 5 d to ensure the circulative period of SRBSDV in the insects [24]. The insects were further confined individually to a healthy rice plant (30 d old) for 2 d and were then detected individually to confirm their SRBSDV infection status. Rice plants exposed to virus-positive insects were maintained for another 15 d and then tested individually for their SRBSDV infection status. Over 15 WBPH females were tested for SRBSDV inoculation in a batch and repeated three times for treatment or control. The SRBSDV inoculation rate was calculated by the number of infected plants in a batch/number of tested plants in the batch.

### 2.5. Data Analysis

The data of qPCR was calculated using the comparative CT method (2^−ΔΔCt^), and normalized against *S. furcifera EF1α*. Analysis of variance (ANOVA) was used to detect the significance of treatment effects on gene expression level and SRBSDV transmission rate. Where there was a significant effect, the Tukey test was used to detect a difference when equal variance was assumed, or Games-Howell test was used when equal variance was not assumed. Virus acquisition and inoculation rates were transformed by the arcsine of square root before ANOVA. The means and standard error (S.E.) shown in the figure were based on untransformed data. All data were analyzed in GraphPad Prism 8.0.

## 3. Results

### 3.1. Bioinformatics Analysis of SfATG3 and SfATG9

Both *SfATG3* (Genbank: OL539544) and *SfATG9* (Genbank: OL539545) are complete-length genes encoding 332 and 764 amino acids, respectively. The identity values of *ATG3* and *ATG9* between *S. furcifera* and *Nilaparvata lugens* are 86.06% and 84.07%, respectively (Table 2).

**Figure 1:**
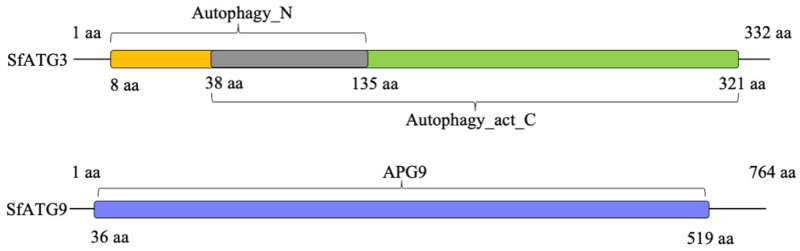
Prediction of the conserved domains of the deduced amino acid sequences of SfATG3 (**upper panel**) and SfATG9 (**down panel**). The autophagy_N domain and autophagy_act_C domain in SfATG3 are indicated in yellow and green boxes, respectively, and the gray box indicates sequences shared by these two domains. The APG9 domain in SfATG9 is indicated in the blue box.

**Figure 2:**
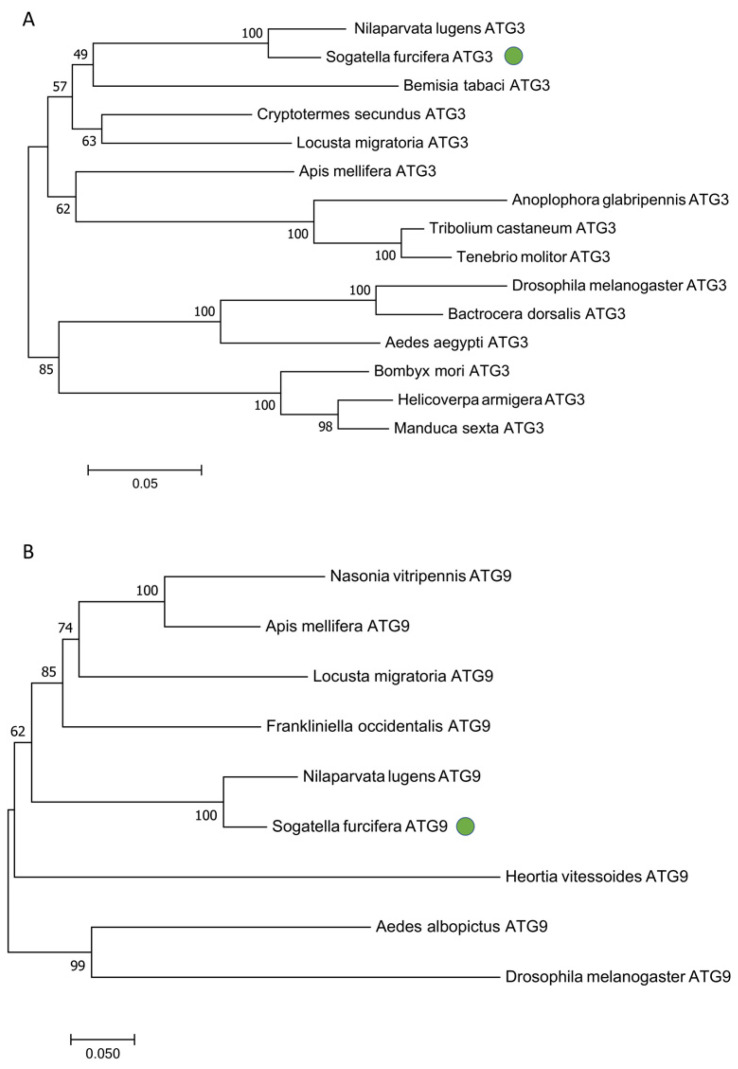
Phylogenetic analysis of ATG3 (**A**) and ATG9 (**B**). Multiple alignments of amino acids of ATG3 and ATG9 were performed by muscle program and the phylogenetic tree was constructed using the neighbor-joining method with a P-distance of MEGA 7.0 (5000 repetitions), respectively.

The NCBI online search tool was used to analyze the conserved domains of the deduced amino acid sequences of *SfATG3* and *SfATG9*, which indicates that *SfATG3* has two conserved domains (Autophagy_N and Autophagy_act_C) and *SfATG9* has one conserved domain (APG9) (Figure 1). The phylogenetic analysis clustered *S. furcifera ATG3* and *ATG9* with *N. lugens ATG3* and *ATG9*, respectively (Figure 2A,B).

### 3.2. Temporal Expression of Virus Gene and Autophagy-Related Genes in SRBSDV-Exposed WBPH Females

**Figure 3:**
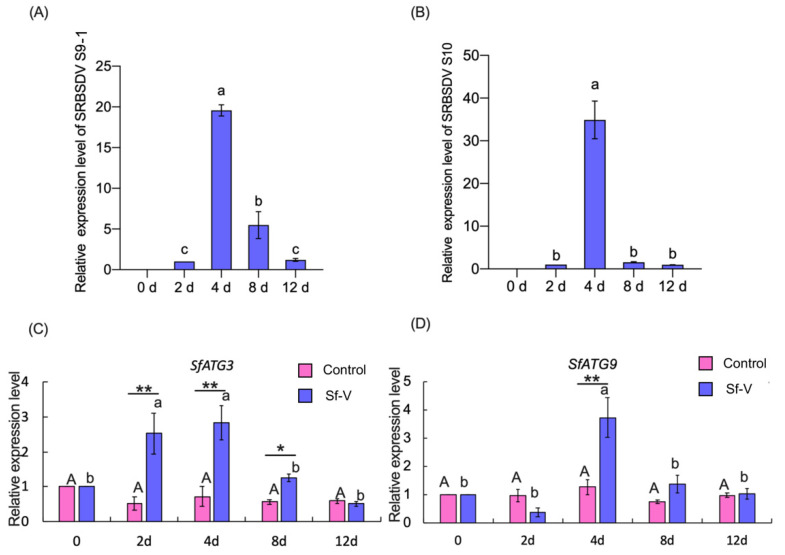
Temporal expression of SRBSDV S9-1 and S10 and autophagy-related genes in response to SRBSDV exposure. (**A**) SRBSDV S9-1. (**B**) SRBSDV S10. (**C**) *SfATG3*. (**D**) *SfATG9.* The expression of SRBSDV S9-1 and S10 was undetectable at 0 dpe. Expression was normalized to the levels of *SfEF1α* that serves as the internal standard. The relative mRNA levels of autophagy-related genes at 0 d and of SRBSDV S9-1 or S10 at 2 d were arbitrarily set to 1. Bars (mean ± S.E.) with different capital or lower-case letters indicate significant differences among times post-exposure (Tukey test, *p* < 0.05). Asterisk indicates significant difference between Sf-V and Control at a certain time post-exposure (*t*-test, *, *p* < 0.05, **, *p* < 0.01). Sf-V: WBPH exposed to SRBSDV-infected rice plants; Control: WBPH exposed to healthy rice plants.

Expression of *SfATG3*, *SfATG9,* and SRBSDV S9-1 and S10 was measured in WBPH females exposed to SRBSDV in comparison to those exposed to healthy rice plants. The expression of SRBSDV S9-1 (Figure 3A) and S10 (Figure 3B) was undetectable in virus-exposed WBPH at 0 dpe, then increased and peaked at 4 dpe and then decreased (F ≥ 58.04; df = 3,8, *p* < 0.001). The temporal expression of the ATG genes followed a similar pattern to that of the SRBSDV S9-1 and S10, also peaking at 4 dpe in SRBSDV-exposed WBPH females (*SfATG3*, F = 8.360, df = 4,10, *p* = 0.003, Figure 3C; *SfATG9*, F = 8.435, df = 4,10, *p* = 0.003, Figure 3D). However, no significant differences in the relative expression levels of the ATG genes in the WBPH exposed to healthy rice plants were detected among different time points (*SfATG3*, F = 2.691, df = 4,10, *p* = 0.093, Figure 3C; *SfATG9*, F = 3.684, df = 4,10, *p* = 0.596, Figure 3D). Expression levels of *SfATG3* and *SfATG9* were significantly higher at 4 dpe and, of *SfATG3* at 8 dpe, in the females exposed to SRBSDV-infected plants (Sf-V) than those to healthy plants (Control) (*t*-test, *p* < 0.05, Figure 3C,D).

### 3.3. SRBSDV Propagation and Transmission by WBPH Treated with dsRNAs

**Figure 4:**
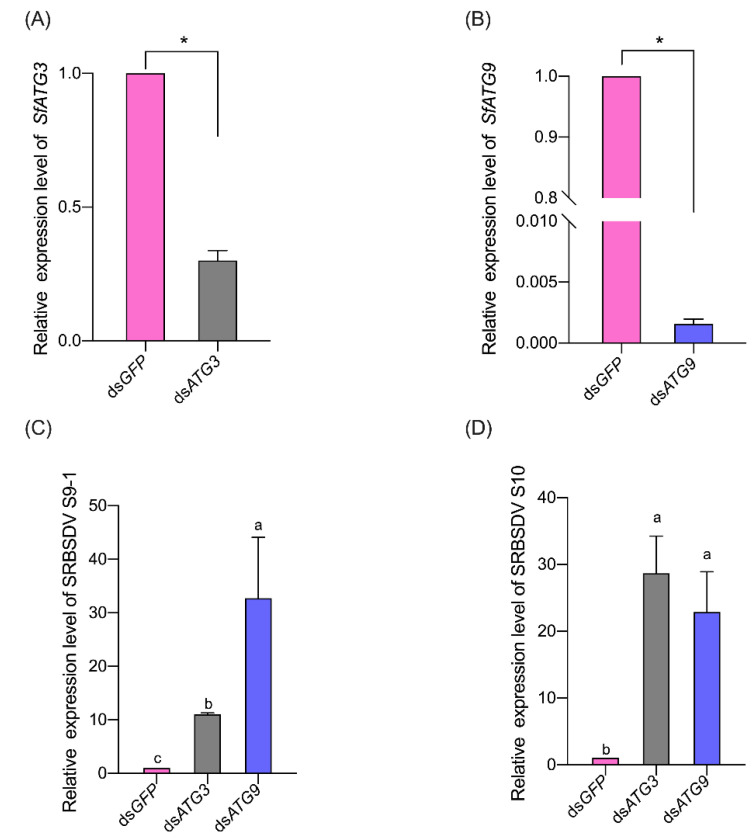
Effects of silencing *SfATG3* or *SfATG9* on the expression of *SfATG3* (**A**) or *SfATG9* (**B**) genes and of SRBSDV S9-1 (**C**) and S10 (**D**). Expression was normalized to the levels of *SfEF1α* that serves as the internal standard. The relative mRNA levels of autophagy-related genes and SRBSDV S9-1 and S10 in ds*GFP*-treated group were arbitrarily set to 1. Bars (mean ± S.E.) with different letters indicate significant differences (Tukey test, *p* < 0.05). Asterisk indicates a significant difference at the 0.05 level between the ds*GFP*-treated group and the ds*ATG3* or ds*ATG9*-treated group (*t*-test).

When the WBPH females were fed with 400 ng/μL (mixed with 15% sucrose, *v/v* = 50:50) ds*ATG3* for 24 h, the expression of *SfATG3* was significantly suppressed in comparison with the insects fed with ds*GFP* (Figure 4A). A similar result was observed for the feeding of ds*ATG9* (Figure 4B). Whereas, feeding WBPH females with 400 ng/μL ds*ATG3* or ds*ATG9* increased the expression of SRBSDV S9–1 and S10 significantly (S9-1, F = 6.080, df = 2,6, *p* = 0.036, Figure 4C; S10, F = 9.513, df = 2,6, *p* = 0.014, Figure 4D). Accordingly, SRBSDV acquisition rate (F = 130.8, df = 2,6, *p* < 0.001, Table 3) and inoculation rate (F = 14.81, df = 2,6, *p* = 0.005, Table 3) were improved significantly in WBPH females fed with 400 ng/μL ds*ATG3* or ds*ATG.* Compared with ds*GFP*, ds*ATG3* and ds*ATG9* treatment improved the SRBSDV inoculation rate by 15.66% (Tukey test, *p* = 0.022) and 22.10% (Tukey test, *p* = 0.004), respectively; and increased SRBSDV acquisition rate by 31.43% (Tukey test, *p* < 0.001) and 35.91% (Tukey test, *p* < 0.001), respectively. The SRBSDV acquisition rate (Tukey test, *p* = 0.231) and inoculation rate (Tukey test, *p* = 0.339) showed no significant difference between ds*ATG3* and ds*ATG9* treatment.

## 4. Discussion

Autophagy can be activated in response to a variety of stimuli, such as viral infection [12,31], among the others. In many plant-virus-vector systems, autophagy is shown to play an antiviral role, while some viruses have evolved to exploit this mechanism to promote their survival, replication, and spread in different ways [33,34]. It was found that the expression of autophagy-related genes correlates with the actual autophagy [21]. The autophagy-related protein ATG3 is essential for autophagosome formation [35,36,37,38], and ATG9 also plays a vital role in autophagy [38,39,40]. In this study, autophagy-related genes, *ATG3* and *ATG9*, were identified in *S. furcifera*. As shown by bioinformatic analysis, the two genes are functionally conserved and homologous to counterparts in *N. lugens*.

In *S. furcifera* exposed to SRBSDV-infected rice plants, the expression of transcript and genomic RNA of SRBSDV S9-1 and S10 displayed a similar unimodal pattern to that of *SfATG3* and *SfATG9* (Figure 3). When the insects are exposed to SRBSDV-infected rice plants, the acquired virus may activate the expression of *SfATG3* and *SfATG9*, which may influence SRBSDV propagation. Therefore, the gene expression is in a unimodal pattern, peaking at 4 dpe. Similar results were observed in the whitefly-TYLCV system, the expression of the TYLCV gene after virus infection also shared a similar pattern to those of autophagy-related genes [16]. When *SfATG3* and *SfATG9* were silenced, SRBSDV propagation and transmission were significantly increased (Figure 4 and Table 3), demonstrating that *SfATG3* or *SfATG9* functions to suppress the propagation and transmission of SRBSDV. Similar results have been reported in the *B. tabaci*-TYLCV system [16]. Therefore, the present results show that the autophagy-related genes *SfATG3* and *SfATG9* function to suppress SRBSDV propagation and transmission. Worthy of mention, a significant difference is observed in SRBSDV S9-1 expression between the ds*ATG3*-treated group and the ds*ATG9*-treated group (Figure 4C), but not in SRBSDV S10 (Figure 4D). Although the specific reasons for such differences are not clear, they can be connected with the differential interactions between the SRBSDV genes (S9-1 and S10) and the ATG genes *SfATG3* and *SfATG9.* Further studies are needed to clarify such interactions. Silencing of ATG genes may alter the feeding behaviors of *S. furcifera*, and feeding behaviors are crucial for virus transmission success [28].

Activation of autophagy in vectors by virus infection has been reported in many vector-virus systems, but plays differential pro- or anti-viral roles. In *B. tabaci*-TYLCV system and *L. striatellus*-rice black steaked dwarf virus (RBSDV) system, autophagy is activated by virus infection or virus protein and functions to suppress the propagation and transmission of the virus [16,17]. However, in the tenuivirus RSV-*L. striatellus* system, virus infection also activates the expression of ATG genes in the vector, but virus infection is promoted instead of suppressed [19]. In RGDV-*R. dorsalis* system, autophagy is also activated by virus infection whereas RGDV propagation in *R. dorsalis* is promoted [18]. From these results, it is obvious that, although the expression of an autophagy-related gene is generally activated in the virus-infected vectors, its pro- or anti-viral role depends on the specific virus-vector system. RSV and RGDV where autophagy plays a pro-viral role are transmitted both horizontally through sap-sucking and vertically through oviposition [41,42,43,44,45], whereas RBSDV and TYLCV where autophagy plays an anti-viral role are transmitted only horizontally through sap-sucking [23,46]. Further studies are needed to generalize the relationship between the role of autophagy and the virus transmission mode and the possible mechanisms behind such a relationship.

In this study, we did not measure autophagy signals and observe autophagic vesicles, as did in previous studies [16,17,18,19]. However, *SfATG3* and *SfATG9* are autophagy-related genes. And our results do show that silencing ATG3 and ATG9 promoted SRBSDV propagation and transmission in the planthopper. It is reasonable to argue that autophagy has occurred in *S. furcifera*. More studies are needed to provide direct evidence showing the role of autophagy in the propagation and transmission of SRBSDV.

Taken together, the current results indicate that autophagy-related genes *SfATG3* and *SfATG9* function to suppress SRBSDV propagation and transmission, which provides a new insight into the control of SRBSDV by using autophagy activator, although there is a long way to go before field application.

## 5. Conclusions

In summary, we identified the autophagy-related genes *ATG3* and *ATG9* from *S. furcifera.* The temporal expression patterns of autophagy-related genes *ATG3* and *ATG9* in *S. furcifera* exposed to SRBSDV-infected rice plants follow that of SRBSDV S9-1 and S10, indicating the propagation of SRBSDV is regulated by autophagy-related genes. The results of *ATG3* and *ATG9* RNAi demonstrate for the first time that autophagy-related genes in WBPH females suppress SRBSDV transmission. Therefore, autophagy-related genes *SfATG3* and *SfATG9* in WBPH play an antiviral role against SRBSDV.

## Figures and Tables

**Table 1:** Sequences of primers used for qPCR of autophagy-related genes and SRBSDV genes S9-1 and S10 and synthesis of ds*GFP*, ds*ATG3,* and ds*ATG9*.

Primers	Sequences (5′-3′)
*SfATG3*_F	CAGGAGATTCCCACACGAATAC
*SfATG3*_R	GTCCTCCTCGTCTAGAAGTCCA
*SfATG9*_F	TCAAAAGGGAACCAGGAGTG
*SfATG9*_R	CTGGCCTGTAAGCTCGATTC
S9-1-F	TCAGAGGTATCAACGGTAGTG
S9-1-R	GTCGGACTTAATAACGCTATCAG
S10-F	CTATGGCGGTTACGACCAAT
S10-R	GACTCCGCTCCATGTTTGTT
*SfEF1α*-F	ATTGTGCTGTGCTGATTGT
*SfEF1α*-R	TGCTCACCTCCTTCTTGAT
ds*ATG3*_F	taatacgactcactatagggACACAAGATGGCATTGAACAAG
ds*ATG3*_R	taatacgactcactatagggTCCTCCTCGTCTAGAAGTCCAC
ds*ATG9*_F	taatacgactcactatagggAGGTTAGGCTGCTTTGTTTTTG
ds*ATG9*_R	taatacgactcactatagggCAATGAATCCATGTTTTTGGTG
ds*GFP*_F	taatacgactcactatagggGGAGAAGAACTTTTCACTGG
ds*GFP*_R	taatacgactcactatagggAGTTGAACGGATCCATCTTC

**Table 2:** Sequence information and alignment of *SfATG3* and *SfATG9*.

Gene	ORF	Full-Length	Sequence Producing Significant Alignment				
			Name	Species	Acc. No.	E-Value	Identity
*SfATG3*	999 bp	Yes	*ATG3*	*N. lugens*	MF040142.1	0	86.06%
*SfATG9*	2295 bp	Yes	*ATG9*	*N. lugens*	MF805755.1	0	84.07%

**Table 3:** Effects of silencing *SfATG3* or *SfATG9* on SRBSDV acquisition and inoculation.

Group	Virus Acquisition Rate (%)	Virus Inoculation Rate (%)
ds*GFP*	26.57 ± 1.64 b	12.40 ± 2.14 b
ds*ATG3*	58.00 ± 1.91 a	28.06 ± 2.32 a
ds*ATG9*	62.48 ± 1.57 a	34.5 ± 4.02 a

Data are expressed as mean ± S.E. Data in a row followed by different letters are significantly different (Tukey test, *p* = 0.05).

## Data Availability

Data available in a publicly accessible repository.

## Supplementary Materials

The following are available online at https://www.mdpi.com/article/10.3390/insects13040394/s1, Figure S1. Agarose gel electrophoresis of dsRNA sequences of *S. furcifera*., Table S1. The GenBank accession numbers of the analyzed *ATG3* sequences in construction of phylogenetic tree; Table S2. The GenBank accession numbers of the analyzed *ATG9* sequences in construction of phylogenetic tree.
