# Supplementary material for: Silencing the Autophagy-Related Genes ATG3 and ATG9 Promotes SRBSDV Propagation and Transmission in Sogatella furcifera
Source: Insects. 2022 Apr 18;13(4):394. doi: 10.3390/insects13040394 (PMC9029546; doi:10.3390/insects13040394)

Table S1. The GenBank accession numbers of the analyzed ATG3 sequences in construction of phylogenetic tree.

	Species name	Accession numbers
**1**	*Drosophila melanogaster*	NP_649059
**2**	*Apis mellifera*	XP_624693
**3**	*Tenebrio molitor*	AHC00664
**4**	*Aedes aegypti*	XP_001657463
**5**	*Helicoverpa armigera*	XP_021197073
**6**	*Bombyx mori*	NP_001135961
**7**	*Tribolium castaneum*	EFA03061
**8**	*Nilaparvata lugens*	AWW05868
**9**	*Locusta migratoria*	ATX63055
**10**	*Manduca sexta*	XP_037301759
**11**	*Bemisia tabaci*	XP_018898322
**12**	*Cryptotermes secundus*	PNF36327
**13**	*Bactrocera dorsalis*	JAC54275
**14**	*Anoplophora_glabripennis*	JAB62976.1

Table S2. The GenBank accession numbers of the analyzed ATG9 sequences in construction of phylogenetic tree.

	Species name	Accession numbers
**1**	*Drosophila melanogaster*	NP_001261023
**2**	*Apis mellifera*	XP_395581.4
**3**	*Aedes albopictus*	XP_001657463
**4**	*Nilaparvata lugens*	AWW05881.1
**5**	*Locusta migratoria*	ATX63059
**6**	*Frankliniella occidentalis*	KAE8750316
**7**	*Nasonia vitripennis*	XP_032457190
**8**	*Heortia vitessoides*	QNO58549.1

Figure S1. Agarose gel electrophoresis of dsRNA sequences of *S. furcifera*

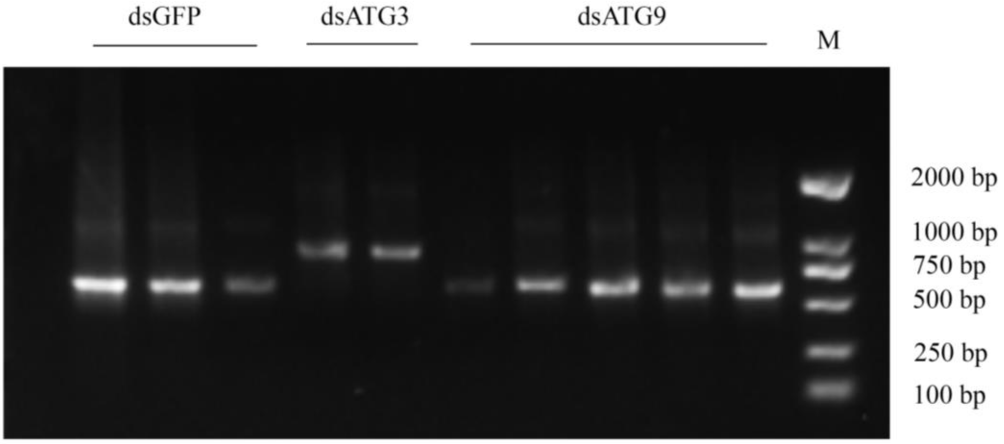

Supplement: Supplementary file 1 [file insects-13-00394-s001.zip › insects-1674353-supplementary.pdf]
